# Transvaginal extraperitoneal single-port laparoscopic sacrocolpopexy for apical prolapse after total/subtotal hysterectomy: Chinese surgeons’ initial experience

**DOI:** 10.1186/s12893-023-02304-z

**Published:** 2024-01-16

**Authors:** Zhiying Lu, Yisong Chen, Chengzhen Xiao, Keqin Hua, Changdong Hu

**Affiliations:** Department of Gynecology, Obstetrics and Gynecology Hospital of Fudan University, 128 Shenyang RD, Shanghai, 200090 China

**Keywords:** Extraperitoneal, Pelvic organ prolapse, Transvaginal natural orifice transluminal endoscopic surgery, Sacrocolpopexy

## Abstract

**Background:**

To introduce a novel technique of transvaginal extraperitoneal single-port laparoscopic sacrocolpopexy (ESLS) for apical prolapse and to evaluate the feasibility and short-term outcomes of this technique.

**Methods:**

Sixteen patients were enrolled to undergo ESLS between January 2020 and May 2021. Perioperative outcomes were included. Short-term results were assessed by Pelvic Floor Distress Inventory-20 (PFDI-20), Pelvic Organ Prolapse Quantification (POP-Q) scores, mesh exposure and prolapse recurrence.

**Results:**

A total of 14/16 cases (87.5%) were successfully completed. The mean operation time was 118 min (range 85–160), and the mean blood loss was 68 ml (range 20–100). The mean postoperative visual analog scale (VAS) pain score at 24 h was 0.7. No intraoperative complications occurred except for one patient who developed subcutaneous emphysema. All patients gained a significant improvement in both physical prolapse and quality of life at 12 months after surgery, and there was no mesh exposure or prolapse recurrence.

**Conclusions:**

Our experience showed that transvaginal ESLS is a feasible and effective technique for apical prolapse with a previous hysterectomy. However, this technique should be performed by surgeons with extensive experience both in vaginal surgery and laparoscopic single-port surgery.

**Supplementary Information:**

The online version contains supplementary material available at 10.1186/s12893-023-02304-z.

## Background

Apical prolapse or vaginal vault prolapse is described as the descent of the vaginal apex after hysterectomy. The incidence is up to 43% [1]. Sacrocolpopexy has become the mainstream surgical method for apical prolapse. The surgical approach is shifted from open to conventional laparoscopy and subsequently to recent transvaginal natural orifice transluminal endoscopic surgery (vNOTES). Without exception, all of the above approaches require entering the pelvic cavity to perform the operation.

The literature shows that 60–90% of patients have different degrees of adhesions after pelvic and abdominal surgery [2]. It was reported that the incidence of postoperative adhesive intestinal obstruction was 5.9‰ after hysterectomy for benign diseases [3]. Postoperative adhesions may lead to the anatomical structural deformation of important organs (such as the bladder, ureter and intestines), and re-entry into the pelvis increases the risk of injury to important organs during secondary operations [4]. Ten broek et al. showed that after previous pelvic and abdominal surgeries, the incidence of intestinal resection was 5.8% at the time of adhesion lysis performed in secondary abdominal surgeries [5]. For patients with previous hysterectomy, it may be a challenge to enter the pelvic cavity through the above three methods for sacrocolpopexy. To avoid risks, we developed an innovative approach for sacrocolpopexy via an exclusive extraperitoneal approach using transvaginal single-port laparoscopy.

The aim of this study was to introduce the novel technique of transvaginal extraperitoneal single-port laparoscopic sacrocolpopexy (ESLS) and to evaluate the feasibility and short-term outcomes of this technique.

## Materials

### Patients and study design

This was a single arm trial at the Obstetrics and Gynecology Hospital of Fudan University, China. The eligibility criteria for ESLS included stage II to IV apical prolapse with previous total/subtotal hysterectomy. All women who met the above criteria between January 2020 and May 2021 were included in this study. The study was approved by our institutional review board (2019-32). All patients gave written informed consent for the surgical procedure and for the use of individual data for research.

The following parameters were included: patient demographics, perioperative outcomes, and short-term results (Pelvic Organ Prolapse Quantification (POP-Q) scores to assess physical prolapse; Pelvic Floor Distress Inventory-20 (PFDI-20) to assess quality of life; mesh exposure and prolapse recurrence). The perioperative data included operative time (from anesthesia to the end of surgery, including other concurrent surgeries), estimated blood loss, surgical complications (injury, blood transfusion, pain, hematoma, infection, and any other complications attributable to the procedure), and recovery of normal diet after operation. Pain was assessed at postoperative 24 h using the visual analog scale (VAS) score: from 0 = no pain to 10 = worst pain. Postoperative follow-up visits were scheduled at 1, 3, 6, and 12 months after surgery.

The POP-Q scores and physical examination were assessed before surgery and at each appointment. Mesh exposure was defined as any mesh that was visible in the vagina on physical examination [6]. Prolapse recurrence was defined as (1) any POP-Q ≥ stage II and (2) any retreatment (pessary or surgery) for prolapse [7].

The PFDI-20 was collected before surgery and at 12 months after surgery. The PFDI-20 includes 3 scales, including the Pelvic Organ Prolapse Distress Inventory 6 (POPDI-6), Urinary Distress Inventory 6 (UDI-6) and Colorectal-Anal Distress Inventory 8 (CRADI-8), with higher total scores indicating a more severe impact of pelvic organ prolapse on quality of life.

### Surgical procedures

All operations were performed by the same medical team who specialized in pelvic floor reconstruction surgery and vaginal surgery and had rich experience in vNOTES in our hospital. The videos showed the surgical techniques. The day before the surgery, the patients received vaginal irrigation without intestinal preparation (such as by drinking laxatives or via an enema to empty the intestinal canal). Prophylactic antibiotics (cefuroxime) were administered 30 min before the surgery. The patient was placed in a lithotomy position to allow exposure for the transvaginal procedure. General anesthesia was performed via endotracheal intubation. A 14 F bladder catheter was inserted to decompress the bladder. In the surgery, a standard rigid 30-degree, 10-mm 3D laparoscope, a single-port device with four trocars (HTKD Med), and 5-mm laparoscopic instruments, including grasping forceps, a needle holder, and an ultrasonic knife (Harmonic), were used.

**Fig. 1:**
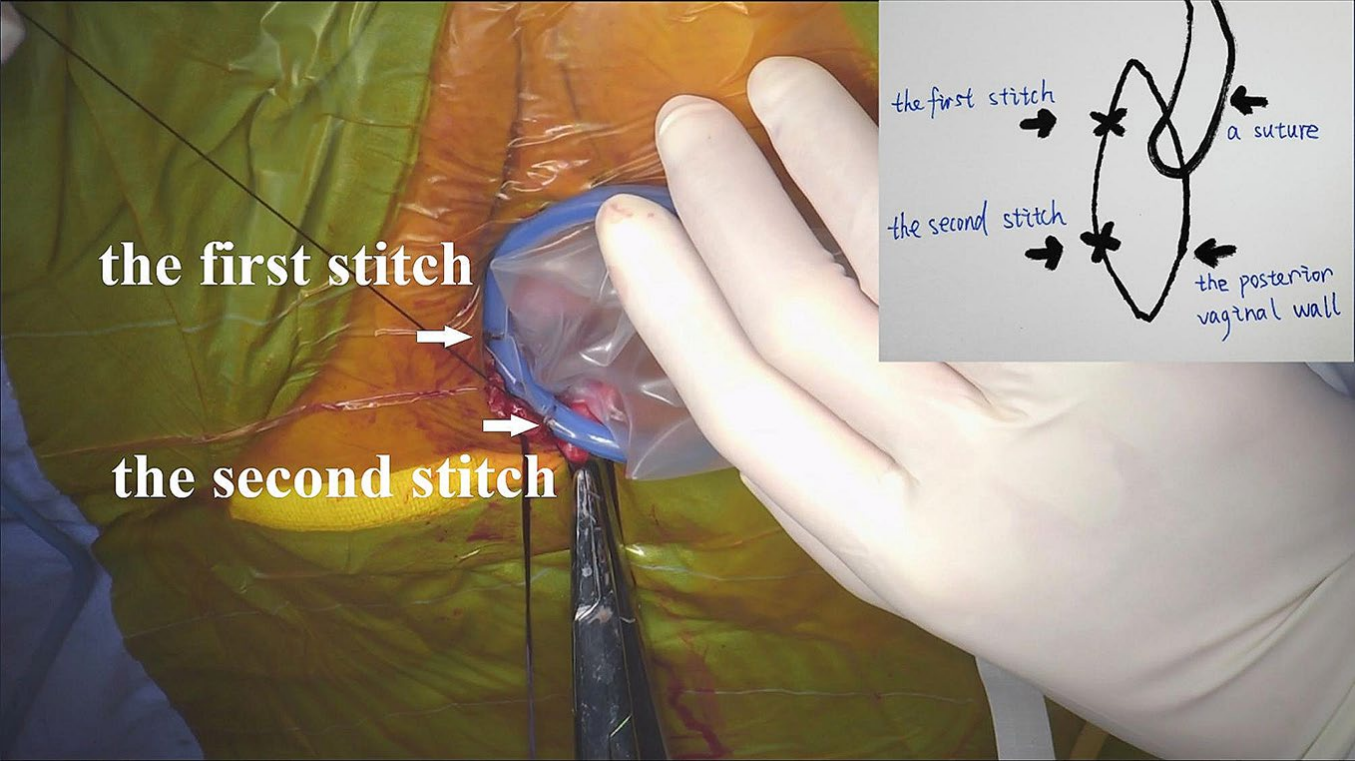
Attach the single-port device to the posterior vaginal wall

For patients with previous subtotal hysterectomy, transvaginal cervical stump resection was first performed. After an injection of methylene blue into the posterior vaginal wall, a 2-cm longitudinal incision was made. The right lateral rectovaginal space was separated with a monopole electric knife through the wound. A transvaginal single-port platform was established. In contrast to conventional vNOTES, the single-port device needs to be sutured to the vaginal wall (Fig. 1). Additionally, a handmade vaginal retractor cut from an oval negative pressure suction ball was used to hold the single-port device.

**Fig. 2:**
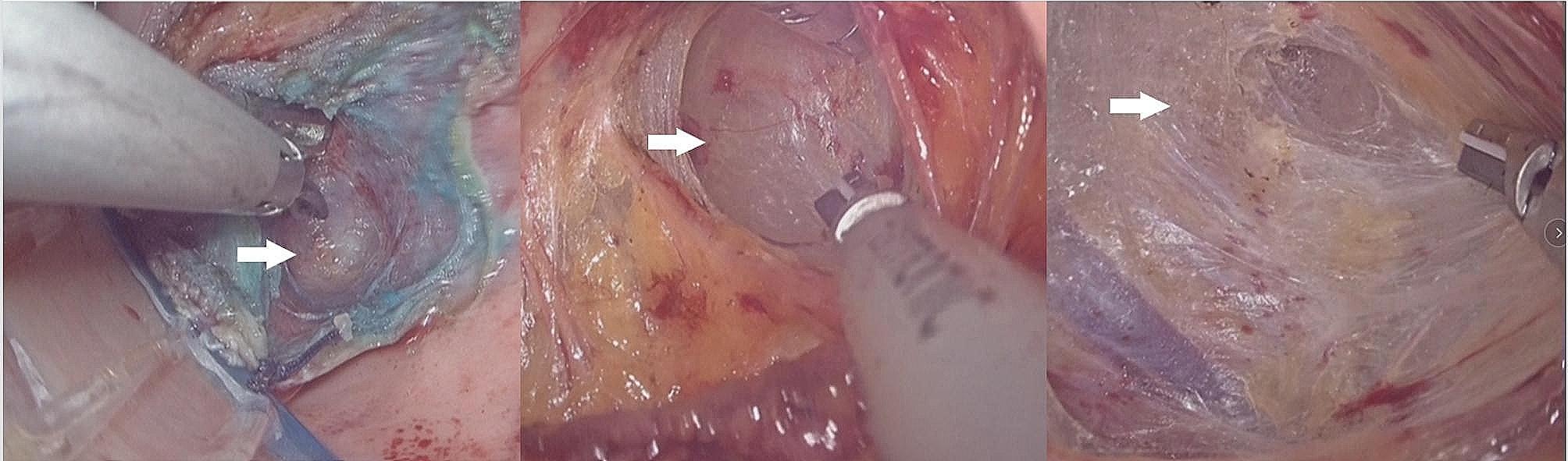
Cotton candy like tissue (white arrow)

**Fig. 3:**
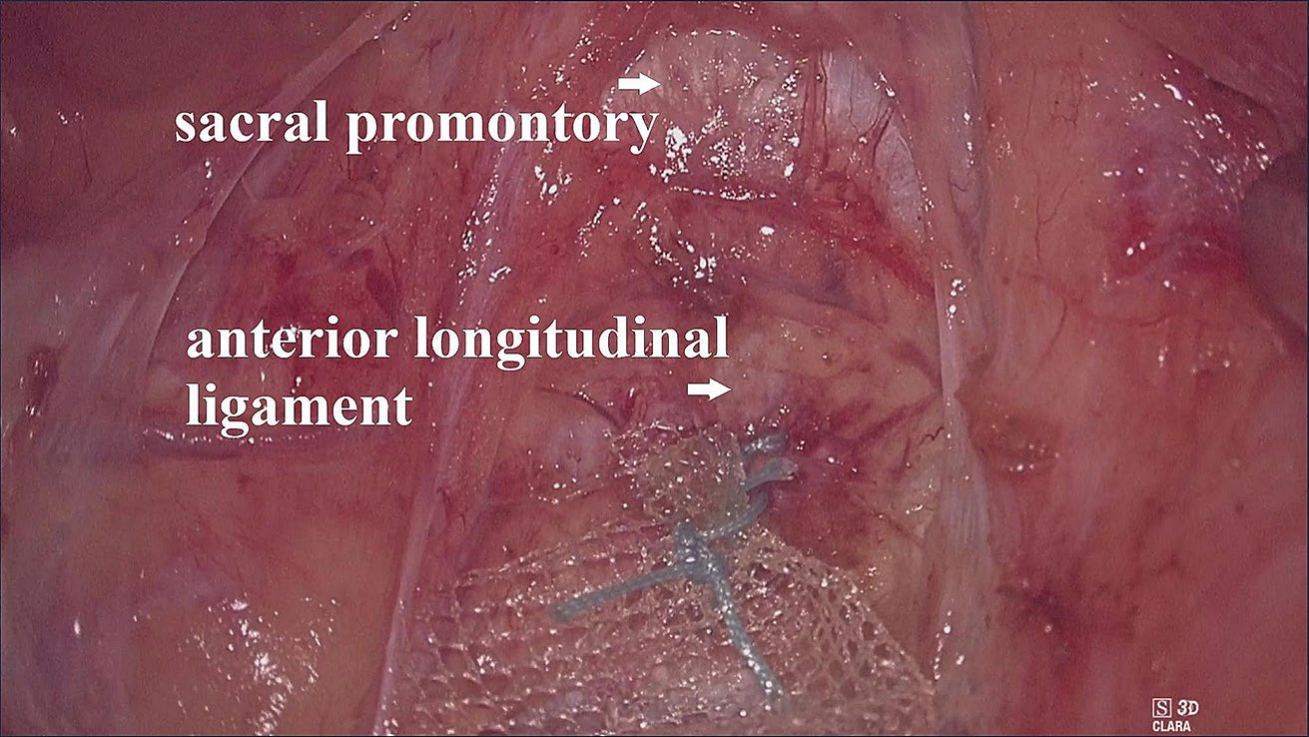
Attach the mesh to the anterior longitudinal ligament below the sacral promontory

The operating table was first adjusted to a position where the patient’s head was low and the patient’s feet were high; then the operating table was adjusted to lean left. The CO_2_ pressure was maintained at 16–18 mmHg. When the ‘cotton candy like tissue’ appeared (Fig. 2), we began to establish the retroperitoneal tunnel. First, the ‘cotton candy like tissue’, actually the retroperitoneal fat or loose connective tissue, was separated upward to the sacral promontory with an ultrasonic knife. In this process, we have to be concerned about the right hypogastric nerve (rHN), right iliac vessels, right ureter, and presacral vessels. After reaching the sacral promontory, a mesh (TiLOOP® Mesh, 6,000,486, pfm medical, Germany) was fixed to the anterior longitudinal ligament on the surface of the first sacral vertebra (S1) with a nonabsorbable suture (Fig. 3). The single-port device was then removed. The mesh was hand sutured to the anterior vaginal wall to suspend the vaginal vault. Before suturing, we measure the length of the vagina assuming the vagina vault is restored, and then tailor the mesh according to the length.

After surgery, cefuroxime was administered once. The normal diet was restored on postoperative day 1. All patients were then followed clinically.

### Statistical analysis

Data collection and statistical analyses were performed using IBM SPSS Statistics 24.0 software (IBM Corp., Armonk, New York, USA). All variables are presented as the mean and standard deviation (SD) or n and percentage (%). Continuous variables were compared by Student’s t test. A P value < 0.05 was considered statistically significant.

## Results

During this period, 16 patients were prepared to undergo transvaginal ESLS. Finally, 2/16 patients (12.5%) were unsuccessfully treated with the technique and converted to vNOTES sacrocolpopexy. In one patient, the peritoneum was opened during the process of establishing the retroperitoneal tunnel. In the other patient, an early accidental peritoneum opening occurred when clamping the vaginal fornix, which was too thin and brittle.

The detailed patient characteristics are listed in Table 1. The mean age was 57 years, and the mean body mass index (BMI) was 23 kg/m^2^. Of the 14 patients, 9 (64.3%) had previous total hysterectomy, and 5 (35.7%) had previous subtotal hysterectomy. Of the 14 patients, 6 (42.9%) had stage II prolapse, 7 (50%) had stage III prolapse and 1 (1.8%) had stage IV prolapse.

**Table 1:** Patient characteristics (n = 14)

Variable	Values
Age (years)	57.6 ± 6.0 (46–70)
Body mass index (kg/m^2^)	23.3 ± 1.6 (21.1–25.6)
Previous total hysterectomy	9 (64.3)
Previous subtotal hysterectomy	5 (35.7)
**Preoperative stage of apical prolapse**	
II	6 (42.9)
III	7 (50)
IV	1 (7.1)

Values are means ± standard deviations (range) or n (%)

The perioperative and short-term outcomes are listed in Table 2. The mean operative time was 118 min, and the mean blood loss was 68 ml. All of the patients had very low postoperative pain scores, with a mean postoperative VAS pain score of 0.7 at 24 h. There were no complications of injury, blood transfusion, hematoma, infection, mesh exposure, or prolapse recurrence. One patient developed subcutaneous emphysema, which spread to the face. It subsided 2 days after the operation. All 14 patients returned to a normal diet on postoperative day 1.

**Table 2:** Perioperative and short-term outcomes (n = 14)

Variable	Values
**Concurrent surgery**	
Cervical stump excision	5 (35.7)
MUS surgery	2 (14.3)
Anterior repair	14 (100)
Posterior repair	12 (85.7)
Perineal body repair	12 (85.7)
Operative time (minutes)	118.9 ± 23.5 (85–160)
Blood loss (ml)	68.6 ± 31.3 (20–100)
**Surgical complications**	
Injury	0
Blood transfusion	0
VAS pain score at 24 h	0.7 ± 0.9 (0–3)
Hematoma	0
Infection	0
Subcutaneous emphysema, spreading to face	1 (7.1)
Recovery of normal diet at the postoperative day 1	14 (100)
Mesh exposure	0
Prolapse recurrence	0

Values are means ± standard deviations (range) or n (%)

MUS: mid-urethral sling

Table 3 shows the changes in the POP-Q scores at 12 months after surgery. The mean pre- and postoperative POP-Q scores were, respectively, + 1.8 ± 1.3 cm and − 2.9 ± 0.4 cm for the Aa point (p = 0.000), + 1.8 ± 2.7 cm and − 7.4 ± 0.7 cm for the C point (p = 0.000), + 7.2 ± 0.4 cm and + 8 ± 0.7 cm for the total vaginal length (p = 0.001), and − 0.6 ± 1.7 cm and − 3.0 ± 0.0 for the Ap point (p = 0.000). All variables showed significant improvement in physical prolapse at 12 months after surgery.

**Table 3:** Change in Pelvic Organ Prolapse Quantification System (POP-Q) score before and after surgery (n = 14)

Case number	Aa		C		TVL		Ap	
	Pre	Post	Pre	Post	Pre	Post	Pre	Post
1	+ 3	−2	+ 2	−7	7	8	0	−3
2	+ 2	−3	−0.5	−6	7	7	0	−3
3	+ 2	−3	+ 4	−7	7	8	0	−3
4	+ 2	−3	−0.5	−7	7	8	−2	−3
5	+ 2	−3	0	−9	7	9	−2	−3
6	+ 3	−3	+ 6	−7	7	8	+ 3	−3
7	+ 2	−3	+ 2	−7	7	8	−2	−3
8	+ 3	−3	+ 7	−8	8	9	+ 3	−3
9	+ 1	−3	+ 3	−7	7	8	0	−3
10	+ 1	−3	+ 4	−8	8	9	−2	−3
11	+ 1	−2	−0.5	−7	8	8	−1	−3
12	+ 3	−3	−1	−8	7	8	−1	−3
13	−2	−3	+ 1	−7	7	7	−2	−3
14	+ 2	−3	−1	−8	7	7	−2	−3
Mean ± standard deviation	+ 1.8 ± 1.3	−2.9 ± 0.4	+ 1.8 ± 2.7	−7.4 ± 0.7	+ 7.2 ± 0.4	+ 8 ± 0.7	−0.6 ± 1.7	−3.0 ± 0.0
P	0.000		0.000		0.001		0.000	

Values are means ± standard deviations. *P < 0.05 was considered statistically significant

TVL: total vaginal length. Pre: preoperative. Post: postoperative

POP-Q score was evaluated at 1 month and 6 months after surgery. These were the latest follow-up examination results of each patient

Table 4 shows the changes in the PFDI-20 scores at 12 months after surgery. The mean pre- and postoperative PFDI-20 scores were 10.3 ± 2.9 and 1.1 ± 2.1 (p = 0.000) for the POPDI-6, 2.5 ± 3.3 and 0.9 ± 2.3 (p = 0.138) for the CRADI-8, 7.9 ± 4.3 and 1.9 ± 2.9 (p = 0.000) for the UDI-6, and 20.6 ± 5.8 and 3.9 ± 5.8 (p = 0.000) for the total PFDI-20. The POPDI-6, UDI-6 and total PFDI-20 scores were significantly decreased after surgery, indicating notable alleviation of the patients’ pelvic and urinary symptoms. The CRADI-8 score did not significantly decrease after surgery, indicating no change in the patients’ colorectal symptoms.

**Table 4:** Change in Pelvic Floor Distress Inventory (PFDI-20) before and after surgery (n = 14)

Case number	PFDI-20 total score		POPDI-6 subscale		CRADI-8 subscale		UDI-6 subscale	
	Pre	Post	Pre	Post	Pre	Post	Pre	Post
1	13	0	9	0	0	0	4	0
2	14	0	10	0	0	0	4	0
3	25	0	12	0	0	0	13	0
4	22	0	9	0	2	0	11	0
5	15	0	6	0	7	0	2	0
6	24	0	12	0	2	0	10	0
7	14	0	9	0	2	0	3	0
8	29	11	17	6	2	0	10	5
9	20	13	7	5	0	0	13	8
10	29	0	15	0	2	0	12	0
11	23	14	9	0	6	8	8	6
12	24	4	10	2	1	0	13	2
13	13	0	10	0	0	0	3	0
14	24	12	9	2	11	4	4	6
Mean ± standard deviation	20.6 ± 5.8	3.9 ± 5.8	10.3 ± 2.9	1.1 ± 2.1	2.5 ± 3.3	0.9 ± 2.3	7.9 ± 4.3	1.9 ± 2.9
P	0.000		0.000		0.138		0.000	

Values are means ± standard deviations. *P < 0.05 was considered statistically significant

Pre: preoperative. Post: postoperative. POPDI-6: Pelvic Organ Prolapse Distress Inventory 6. CRADI-8: Colorectal Anal Distress Inventory 8

UDI-6: Urinary Distress Inventory 6

## Discussion

Laparoscopic minimally invasive surgery through the extraperitoneal approach has been applied for lymph node dissection in cases of gynecologic carcinoma [8, 9], colorectal resection [10], nephrectomy [11] and adrenalectomy [12] but has never been used for sacrocolpopexy. This is the first study of transvaginal ESLS for patients with apical prolapse and a previous hysterectomy. Significant improvements in both physical prolapse and quality of life with few complications suggest that transvaginal ESLS may be feasible and effective.

The sacrocolpopexy via an extraperitoneal approach has some potential advantages. First, it is not required to enter the pelvic cavity during the extraperitoneal approach. Women with previous pelvic surgery often have postoperative adhesions [13]. Re-entering the pelvic cavity in subsequent surgery is more difficult and is associated with a higher risk of morbidity and mortality, inability to perform laparoscopic surgery and conversion to laparotomy and injury to organs such as the small bowel, bladder or ureters [14, 15]. Our preliminary results are encouraging, and there were no intraoperative injuries in this study.

Second, the operation does not involve the small intestine or sigmoid colon, which accelerates the patient’s recovery. In routine operations through a laparoscopic transperitoneal approach (via transumbilical or transvaginal), it is necessary to manipulate the small intestine and sigmoid colon through instruments to expose the presacral region. In patients with adhesions, it is necessary to separate the adhesions and then push away the intestine. Direct manipulation of the intestine increases the risk of intestinal injuries, postoperative intestinal obstruction and pain [16]. Our preliminary results are gratifying, with low postoperative pain and a quick return to a normal diet. In the course of our procedure, the surgical field of view is exposed in the following two ways. (1) By adjusting the patient’s position, the intestine and omentum fall away from the pelvis naturally to reduce the pressure on the peritoneum in front of the presacral region. (2) It increases the retroperitoneal pneumoperitoneum pressure and separates the retroperitoneal fat or loose connective tissue to expand the surgical field.

This procedure has some technical difficulties and limitations. First, the main difficulty of the approach is the establishment of a retroperitoneal tunnel because there is no fixed anatomical mark for guidance. If lucky enough, surgeons could identify the right hypogastric nerve (supplementary video) or ureter included in fat or loose connective tissue during the process of establishing the retroperitoneal tunnel from the rectovaginal space to the presacral space below the sacral promontory. These important anatomical markers point in the right direction. However, sometimes the anatomical markers are not obvious, and there is only ‘cotton candy like tissue’ (supplementary video). Depending on their experience, surgeons should separate the ‘cotton candy like tissue’ upward to the sacral promontory. Some technical precautions are required to establish the retroperitoneal tunnel with the ‘cotton candy like tissue’. (1) When separating the right lateral rectovaginal space at the very beginning of the surgery, it is not recommended to separate the tissues too deeply, as this will destroy the ‘cotton candy like tissue’. (2) Airtightness and no air leakage should be ensured after the establishment of a single-port platform. To achieve this, the single-port device was sutured to the vaginal wall, and a handmade vaginal retractor was used to avoid it slipping away from the vagina. (3) It is necessary to maintain a sufficiently high retroperitoneal pressure to form visible ‘cotton candy like tissue’ and create a good visual field.

Second, surgeons with extensive experience in vaginal surgery and laparoscopic single-port surgery are needed. The operative space is very limited and is much smaller than that of vNOTES. In this study, although challenging, the technical difficulties did not compromise the safety and effectiveness of the procedure.

Third, this technology also brings new problems: (1) High CO_2_ pressure easily causes subcutaneous emphysema and even spreads to the face and neck. In our early study, one patient developed the above condition, which subsided 2 days after the operation. (2) The patients were in a head-down position during the operation, which resulted in an increase in the intracranial and ocular pressure [17], which limits the application of this procedure in patients with severe craniocerebral disease and/or with glaucoma. (3) An unexpected peritoneum opening may injure the intestine on the other side of the peritoneum during the establishment of the retroperitoneal tunnel. Adjusting the position of the operating table during the operation may reduce this risk.

The limitations to this study include the small sample size from a single center because this was a pilot evaluation of a new surgical technique. Additionally, the follow-up duration was short.

## Conclusions

Transvaginal ESLS is a feasible and effective method to manage patients with apical prolapse and a previous hysterectomy. Overall, this process decreases the risk of intraperitoneal organ injury and reduces the impact on intestinal function. However, this technique should certainly be performed by surgeons with extensive experience in vaginal surgery and laparoscopic single-port surgery.

### Electronic supplementary material

Below is the link to the electronic supplementary material.

**Supplementary Material 1: Supplementary Video:** Transvaginal extraperitoneal single-port laparoscopic sacrocolpopexy for apical prolapse after total hysterectomy

**Supplementary Material 2: Supplementary Video:** The right hypogastric nerve, as an anatomical marker in establishing the retroperitoneal tunnel

## Data Availability

The datasets used and analyzed during the current study available from the corresponding author on reasonable request.

## Abbreviations

vNOTES: transvaginal natural orifice transluminal endoscopic surgery
ESLS: transvaginal extraperitoneal single-port laparoscopic sacrocolpopexy (ESLS)
POP-Q: Pelvic Organ Prolapse Quantification
PFDI-20: Pelvic Floor Inventory-20
VAS: visual analog scale
POPDI-6: Prolapse Distress Inventory 6
UDI-6: Urinary Distress Inventory 6
CRADI-8: Colorectal-Anal Distress Inventory 8
rHN: right hypogastric nerve
S1: the first sacral vertebra
